# Perspectives in computational mass spectrometry: recent developments and key challenges

**DOI:** 10.1093/bioadv/vbaf301

**Published:** 2025-12-17

**Authors:** Timo Sachsenberg, Lindsay K Pino, Marie Brunet, Isabell Bludau, Oliver Kohlbacher, Juan Antonio Vizcaino, Wout Bittremieux

**Affiliations:** Department of Computer Science, University of Tübingen, Tübingen, 72074, Germany; Institute for Bioinformatics and Medical Informatics, University of Tübingen, Tübingen, 72074, Germany; Talus Bio, Seattle, WA 98122, United States; Pediatrics Department, University of Sherbrooke, Sherbrooke, Québec J1K 2R1, Canada; Department of Neuropathology, Institute of Pathology, University Hospital Heidelberg, Heidelberg, 69120, Germany; Department of Computer Science, University of Tübingen, Tübingen, 72074, Germany; Institute for Bioinformatics and Medical Informatics, University of Tübingen, Tübingen, 72074, Germany; Translational Bioinformatics, University Hospital Tübingen, Tübingen, 72074, Germany; European Molecular Biology Laboratory, European Bioinformatics Institute (EMBL-EBI), Hinxton CB10 1SD, United Kingdom; Department of Computer Science, University of Antwerp, Antwerp, 2020, Belgium

## Abstract

**Summary**: Mass spectrometry (MS) is a cornerstone technology in modern molecular biology, powering diverse applications across proteomics, metabolomics, lipidomics, glycomics, and beyond. As the field continues to evolve, rapid advancements in instrumentation, acquisition strategies, machine learning, and scalable computing have reshaped the landscape of computational MS. This perspective reviews recent developments and highlights key challenges, including data harmonization, statistical confidence estimation, repository-scale analysis, multi-omics integration, and privacy in clinical MS. We also discuss the increasing importance of machine learning and the need to build corresponding literacy within the community. Finally, we reflect on the role of the Computational Mass Spectrometry (CompMS) Community of Special Interest of the International Society for Computational Biology in supporting collaboration, innovation, and knowledge exchange. With MS-based technologies now central to both basic and translational research, continued investment in robust and reproducible computational methods will be essential to realize their full potential.

## 1 Introduction

Mass spectrometry (MS) is a central analytical technology in molecular biology, widely used to characterize the molecular composition of complex biological systems. Its capacity to measure the mass-to-charge ratio of ions with high sensitivity underpins its widespread adoption across diverse omics disciplines—including proteomics, metabolomics, lipidomics, and glycomics—to investigate phenotypes in both health and disease.

Ongoing advances in MS instrumentation and computational methods have significantly expanded the scale and resolution of MS-based experiments. These developments have enabled emerging applications such as single-cell proteomics, spatially resolved molecular profiling, and high-throughput clinical studies. In parallel, computational MS has evolved to address increasingly complex tasks, including real-time data interpretation, machine learning (ML)-driven molecular property prediction, and large-scale integration across datasets.

However, the growing volume and complexity of MS data also introduce new challenges. Scalable computational infrastructure is needed to support high-throughput workflows. Data harmonization across instruments, batches, and modalities remains difficult, especially when integrating multi-omics data. While proteomics has developed advanced methods for statistical confidence estimation, challenges persist for non-standardized applications. In contrast, metabolomics often lacks similar mature statistical approaches, making confidence assessment even more difficult in this domain. Additionally, emerging concerns about data privacy in clinical applications, alongside the need for robust metadata standards and FAIR (Findable, Accessible, Interoperable, and Reusable) (Wilkinson *et al.* 2016) compliance, further underscore the need to address infrastructural and ethical dimensions.

In this perspective, we provide an overview of major recent developments in computational MS, outline key challenges facing the field, and discuss emerging opportunities for continued innovation. We also highlight the role of the Computational Mass Spectrometry (CompMS) Community of Special Interest of the International Society for Computational Biology (ISCB) in fostering collaboration, innovation, and community building. As MS continues to support increasingly diverse and complex omics applications, the development of robust, scalable, and transparent computational methods will be essential to ensure accurate data interpretation and meaningful biological insight.

## 2 Recent developments in computational mass spectrometry

### 2.1 Emergence of novel mass spectrometry instrumentation

Recent advances in MS instrumentation have substantially improved sensitivity, speed, and analytical depth across a range of applications. Instruments such as Thermo Fisher Scientific’s Astral (Heil *et al.* 2023), Sciex’s ZenoTOF (Wang *et al.* 2022), and Bruker’s timsTOF (Demichev *et al.* 2022) now support high-throughput workflows capable of generating tens of gigabytes of data per instrument hour. While these capabilities enable deeper interrogation of biological systems, they also present considerable challenges in data handling, necessitating scalable solutions for storage, processing, and downstream analysis.

To manage increasing data volumes, the development of computational tools optimized for efficiency has become essential. In proteomics, software such as MSFragger (Kong *et al.* 2017) and Sage (Lazear 2023), and in metabolomics, tools like Clustering+/Networking+ (Mongia *et al.* 2024) and mzMine (Schmid *et al.* 2023), exemplify recent efforts to support high-throughput analysis at scale, reflecting the broader trend toward improved performance and scalability in computational MS. However, meeting the demands of modern instrumentation requires more than faster algorithms; it calls for rethinking entire analysis workflows to ensure that they can accommodate heterogeneous data structures and acquisition modes while maintaining reproducibility and interoperability.

A major enabler of such interoperability has been the vendor-agnostic mzML format (Martens *et al.* 2011), which provides a universal interface between raw instrument data and downstream computational tools. This openness allows researchers to apply shared analysis pipelines regardless of the instrument manufacturer, supporting reproducibility and the development of cross-platform software ecosystems. Yet, rapid technological innovation continually tests the flexibility of this standard. For example, the growing use of ion mobility spectrometry adds an additional analytical dimension that enhances molecular separation but greatly increases data volume and complexity. Likewise, the expanding range of fragmentation techniques, including electron-transfer dissociation and ultraviolet photodissociation in addition to conventional collision-induced and higher-energy collisional dissociation, requires software to adapt to distinct fragmentation behaviors and scoring models. Together, these advances create a moving target for developers, who must continually update algorithms and data models to remain compatible with emerging instrument capabilities.

At the same time, the efficiency of underlying data storage formats has become a growing concern. The mzML format remains the primary open format for MS data exchange, but its XML-based structure can lead to file sizes an order of magnitude larger than the corresponding vendor raw files. Moreover, mzML is not natively compatible with modern data science frameworks, requiring specialized parsers for access and manipulation. To address these limitations, the community is exploring more compact and computation-friendly alternatives, such as the proposed “mzPeak” format (Van Den Bossche *et al.* 2025), which leverages a columnar Parquet-based design to improve compression, query performance, and interoperability with contemporary data science tools.

Beyond computation, the growing size of raw MS data and experiment sample size imposes logistical and financial pressure on infrastructure. Laboratories must find cost-effective strategies for data storage, transfer, and archival, particularly as datasets accumulate over time. Approaches such as spectrum clustering to reduce redundancy (Griss *et al.* 2016) and numerical compression techniques (Teleman *et al.* 2014) have shown promise in limiting storage overhead while preserving analytical utility. Continued investment in such methods is essential to ensure that advances in instrumentation remain accessible and sustainable across diverse research settings.

### 2.2 Advances in mass spectrometry data acquisition

Recent developments in MS data acquisition strategies are expanding the types of biological questions that can be addressed, while also placing new demands on computational analysis. One major trend is the emergence of techniques for low-input and single-cell proteomics, which enable molecular profiling at the level of individual cells (Bennett *et al.* 2023). These approaches rely on sensitive instrumentation and require computational frameworks capable of addressing the variability and sparsity inherent to single-cell data. As single-cell MS becomes more widely adopted, bioinformatics tools must evolve to support reproducible and statistically robust analyses at this resolution.

In parallel, spatial approaches, including MS imaging, are gaining traction in both proteomics (Method of the Year 2024: spatial proteomics 2024), metabolomics (Wadie *et al.* 2024), and lipidomics. These spatial techniques contribute crucial insights into the localization and abundance of molecules within biological tissues, enhancing our understanding of molecular organization within complex biological structures. Recent developments such as deep visual proteomics (Mund *et al.* 2022) have demonstrated the potential of spatially resolved MS for clinical research, enabling molecular characterization of tissue architecture at near-cellular resolution and revealing disease-associated spatial phenotypes. A related and promising direction lies in integrating MS-based measurements with other imaging modalities, for example combining protein–protein interaction data from BioPlex (Huttlin *et al.* 2015, 2017) with fluorescence-based localization data from the Human Protein Atlas (Uhlen *et al.* 2010) to uncover new molecular relationships within subcellular systems (Qin *et al.* 2021). Collectively, these multimodal and spatially informed strategies hold considerable promise for advancing systems-level biology. However, the need to analyze large, multidimensional datasets calls for robust computational solutions that can handle both the volume and specificity of spatial data. As spatial resolution and data complexity continue to increase, scalable and domain-specific bioinformatics tools are needed to manage data volume, integrate data across modalities, and extract biologically meaningful spatial patterns. Developing such frameworks, capable of reconciling heterogeneous data types and scales, remains a central open challenge.

The increasing mass range of MS instruments has enabled the data acquisition of intact proteins without prior digestion [top-down proteomics (Roberts *et al.* 2024)]. The resulting data exhibit different characteristics compared to classical bottom-up proteomics and offer more comprehensive insights into individual proteoforms (Marx 2024). Fully interpreting these data requires specialized algorithms and software capable of deconvolving complex isotope distributions and charge states in top-down spectra, as well as accurate identification and quantification of proteoforms (Kou *et al.* 2016, Jeong *et al.* 2020). Moreover, representing the resulting proteoform-level information necessitates standardized formats that capture this added molecular complexity, such as the ProForma notation (LeDuc *et al.* 2022).

Another trend in MS data acquisition is the emphasis on high-throughput acquisition. Strategies combining short chromatographic gradients with fast scanning instruments enable the analysis of large sample cohorts, including clinical and pharmacological studies (Messner *et al.* 2020). While this facilitates more comprehensive experimental designs, it also shifts the computational bottleneck downstream, requiring highly automated and efficient data processing pipelines to keep pace with acquisition.

In addition, specialized techniques such as real-time searching (Schweppe *et al.* 2020, Bills *et al.* 2022) and advanced data acquisition methods (Mendes *et al.* 2024) are being adopted to increase experimental robustness and depth. Real-time search engines provide immediate feedback during acquisition, while complex data-independent (Mendes *et al.* 2024) and iterative acquisition (Zuo *et al.* 2021) approaches aim to reduce sampling bias and improve reproducibility.

Overall, the increasing diversity and complexity of MS acquisition strategies call for equally flexible computational tools. Although existing software often provides a starting point, data generated by cutting-edge methods frequently necessitates the development of new algorithms and data models tailored to their specific characteristics. Addressing these needs will be key to ensuring that technological advances in MS acquisition are matched by reliable and scalable computational interpretation.

### 2.3 Demand for enhanced computational resources

The growing scale and complexity of MS experiments are placing increasing demands on computational infrastructure. Traditionally, many laboratories have relied on local compute workstations or institutional high-performance computing (HPC) clusters for data analysis. However, with modern instruments producing increasingly large and information-rich datasets, these resources are often pushed to their limits. The need for scalable solutions spans not just data processing, but also storage, retrieval, and integration across multiple projects and platforms.

Scalability in computational MS encompasses three interrelated dimensions: algorithmic, systems, and operational scalability. First, algorithmic scalability refers to the efficiency of the computational methods themselves. Tools must be designed to handle rapidly growing data volumes through more efficient algorithms and parallelization strategies. Recent progress in advanced fragment-ion indexing approaches (Kong *et al.* 2017, Lazear 2023, Mongia *et al.* 2024, Li and Fiehn 2023) exemplifies how algorithmic innovation can drastically accelerate spectral matching and reduce computational overhead. Continued development of such methods will be essential to ensure that gains in instrumentation throughput are matched by corresponding improvements in computational performance.

Second, systems scalability involves the ability of computational infrastructure to accommodate expanding workloads and data sizes. Cloud-based computing environments have emerged as a flexible complement to local infrastructure (Neely 2021). These platforms offer access to specialized hardware, such as graphics processing units and high-memory nodes, that are well-suited for intensive tasks like ML, large-scale (re)analysis, and complex statistical modeling. Their scalability allows users to allocate resources dynamically based on project needs, and the pay-as-you-go model can help manage costs. Increasingly, cloud solutions are becoming a key enabler of reproducible, high-throughput MS workflows that can scale seamlessly from individual projects to community-wide data reanalyses.

Third, operational scalability concerns the reproducibility, portability, and maintainability of computational workflows across different environments and laboratories. Achieving this requires transparent, containerized, and workflow-managed solutions, for example built on Nextflow (Di Tommaso *et al.* 2017), that allow analyses to be executed consistently regardless of local infrastructure. This form of scalability ensures that pipelines developed in one laboratory can be deployed in another without modification, facilitating collaboration and cross-institutional benchmarking. Operational scalability thus bridges algorithmic advances and systems capacity, enabling reproducible science at scale.

ML plays an increasingly important role across many stages of MS data analysis. In proteomics, ML models are now widely used to predict peptide properties such as retention time (Gessulat *et al.* 2019, Bouwmeester *et al.* 2021, Zeng *et al.* 2022), ion mobility (Zeng *et al.* 2022, Devreese *et al.* 2025), and fragment ion intensities (Degroeve and Martens 2013, Gessulat *et al.* 2019, Zeng *et al.* 2022), increasing sensitivity by enhancing downstream tasks like spectrum matching and rescoring (Kalhor *et al.* 2024). Tools such as Percolator (Käll *et al.* 2007) integrate these predictions to improve peptide–spectrum match confidence. ML is also advancing *de novo* peptide sequencing (Bittremieux *et al.* 2024), with recent models achieving substantial improvements over traditional algorithms. By automating aspects of data analysis and improving the interpretability of MS results, ML has become a key asset in modern proteomics.

In metabolomics, ML applications are emerging but are not yet as mature. The chemical diversity and limited ground truth annotations in metabolomics create unique challenges. Nonetheless, progress is being made in tasks such as spectrum modeling (Wang *et al.* 2021, Young *et al.* 2024), analog detection (Huber *et al.* 2021), *in silico* database searching (Dührkop *et al.* 2019, Goldman *et al.* 2023), and *de novo* annotation of unknown compounds (Stravs *et al.* 2022). A key limiting factor remains the availability of high-quality benchmark datasets. While proteomics has benefited from large public training sets, equivalent resources in metabolomics have only recently started to emerge. Notably, MassSpecGym provides a benchmark suite to facilitate model evaluation and development (Bushuiev et al. 2024), helping to advance ML applications in this domain.

A promising direction for future work is the development of foundation models trained on large-scale MS data. Inspired by advances in other omics domains, these models are pre-trained on broad datasets and can be fine-tuned for specific downstream applications (Sanders *et al.* 2025, Bushuiev *et al.* 2025). Initial foundation models for MS have focused on spectral data, but future iterations could incorporate additional context such as metadata, literature, or a broader representation of molecular complexity across different omics layers. These models have the potential to support cross-domain applications in proteomics, metabolomics, and beyond, providing a unified framework for interpreting complex MS data.

## 3 Challenges and opportunities in computational mass spectrometry

**Figure 1.**
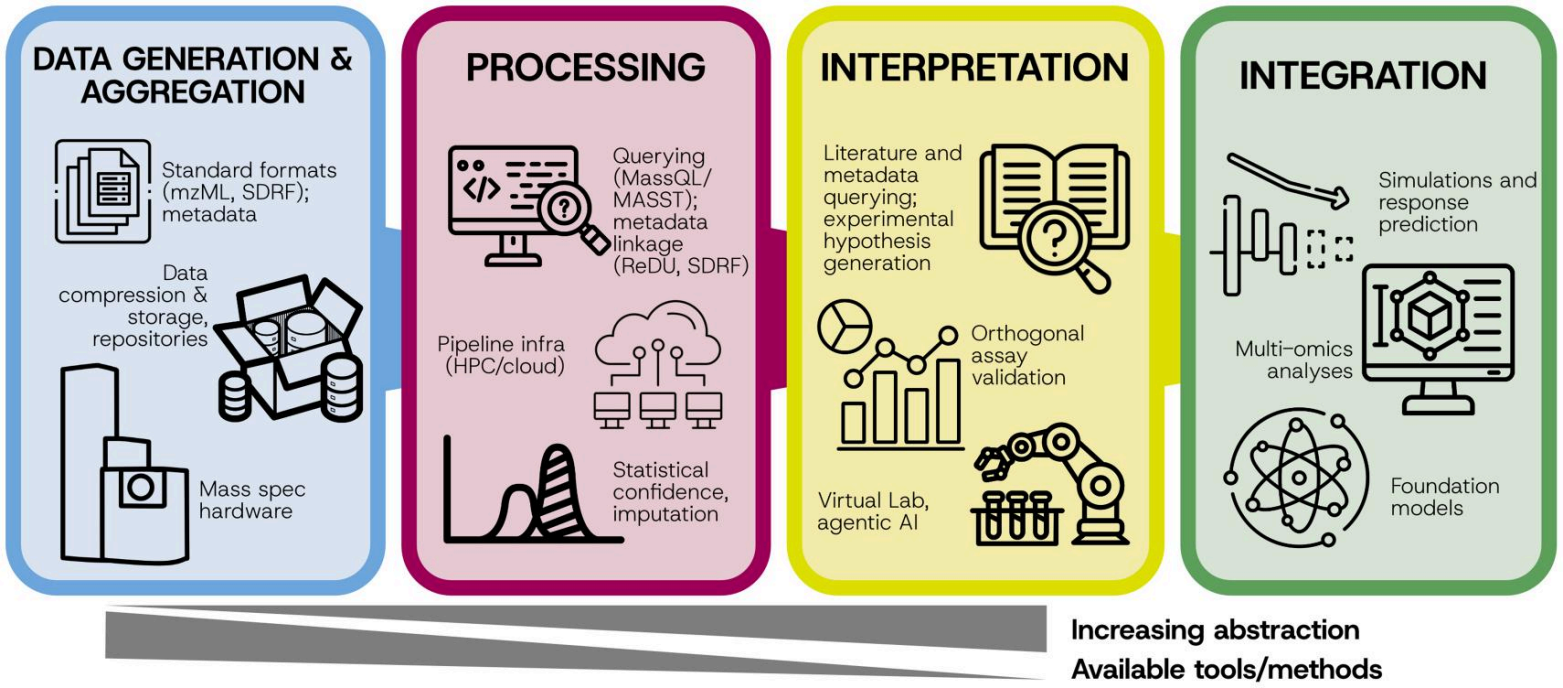
Computational MS spans a continuum from raw data generation to systems-level integration. Key challenges and opportunities arise at each stage of the workflow, from hardware and data formats, to cloud-scale processing, statistical confidence, interpretation frameworks, and multi-omics integration. Progress along this pipeline creates new opportunities for innovation, including virtual labs, agentic AI, and foundation models that can enable more autonomous and integrative data interpretation.

In this section, we outline major bioinformatics challenges and highlight emerging opportunities across the computational MS landscape (Fig. 1).

### 3.1 Data harmonization across experiments and modalities

Heterogeneity in MS data remains a major obstacle to integrative analysis. Differences in instrumentation, acquisition settings, and experimental protocols—both within and across laboratories—can introduce systematic variation that complicates comparisons. Even repeated measurements on the same instrument can yield different results due to temporal shifts in sensitivity, ionization efficiency, or calibration (Bittremieux *et al.* 2018). This variability becomes particularly problematic when aggregating datasets from multiple sources, where technical artifacts may mask or confound underlying biological signals.

Among the most persistent sources of technical variation are batch effects, which can obscure genuine biological differences and hinder the comparability of datasets across studies and time points (Leek *et al.* 2010). While batch correction methods exist and are routinely used in both proteomics and metabolomics, they are often optimized for relatively homogeneous datasets. In more complex settings, such as multi-center studies or retrospective analyses (Tabb *et al.* 2010), current methods may fail to remove technical variation without also suppressing biological structure. More robust, data-driven methods are needed to support harmonization in increasingly complex MS applications, including multi-center, longitudinal, and multi-omics studies.

In related omics fields such as genomics and transcriptomics, substantial progress has been made in mitigating batch effects through various approaches such as empirical Bayes modeling (Johnson *et al.* 2007) and manifold alignment (Korsunsky *et al.* 2019). These frameworks have proven effective in normalizing large, multi-batch datasets, including single-cell transcriptomic studies, by aligning shared biological signals while reducing technical variance. However, the challenge is even greater for MS-based omics, where data are inherently noisier, feature detection and quantification are less standardized, and measurement variability can be introduced at multiple stages of the workflow. Consequently, many methods that can succeed in genomics domains do not transfer straightforwardly to MS data, where missingness, non-linear signal distortion, and feature overlap complicate direct correction.

Addressing these challenges is central to realizing heterogeneous data integration in MS-based omics. Without effective batch harmonization, datasets from different laboratories, instruments, or acquisition protocols remain only partially comparable, limiting opportunities for repository-level meta-analysis and ML model training. Overcoming this limitation would enable disparate experiments to contribute to a shared, cumulative understanding of biological systems, reducing the need for redundant experiments and accelerating discovery through reanalysis of existing data resources.

Deep learning offers a potential path forward. With the ability to learn complex, non-linear relationships across datasets, neural networks have been proposed as tools for data harmonization and imputation (Webel *et al.* 2024). Initial applications suggest that deep learning can model batch variation while preserving signal, even in the presence of missing data and instrument-specific noise. If trained on diverse and well-annotated datasets, such models could enable reuse of previously incompatible data, enhancing the utility of existing MS repositories. However, these methods must be applied with care, as overly aggressive batch correction can inadvertently suppress biologically meaningful variation or rare phenotypes. Rigorous validation and benchmarking across independent datasets are therefore essential to ensure that batch correction improves data comparability without obscuring true biological differences.

Beyond harmonization within a single omics modality, the field is also moving toward multi-modal data integration. In proteomics, combining MS-based measurements with non-MS, affinity-based technologies such as Olink or SomaLogic can increase coverage and sensitivity (Beimers *et al.* 2025). While these approaches are particularly useful in large cohorts (Ferkingstad *et al.* 2021, Sun *et al.* 2023), they cannot provide information on post-translational modifications (PTMs) and protein isoforms, where MS retains a clear advantage. In metabolomics, combining MS data with nuclear magnetic resonance (NMR) spectroscopy can improve structural elucidation and quantification. Despite the complementarity of these technologies, their integration is nontrivial. Differences in resolution, dynamic range, data structure, and software pipelines pose significant barriers. Addressing these will require not only computational innovation but also greater standardization in data formats and metadata reporting across platforms.

### 3.2 Statistical confidence for robust data interpretation

Reliable statistical assessment is fundamental to interpreting MS data with confidence. Among the most widely used approaches, false discovery rate (FDR) estimation (Storey 2011) plays a central role in distinguishing true positives from random matches. Accurate FDR control is critical for ensuring reproducibility, enabling cross-study comparisons, and building trust in downstream biological conclusions.

In proteomics, FDR estimation methods are well established, particularly through the use of target–decoy competition strategies (Elias and Gygi 2007). These methods have become integral to peptide and protein identification pipelines, allowing researchers to systematically control the rate of false positives in large proteomic datasets. Nevertheless, proper FDR estimation remains a complex and nuanced challenge, and even subtle methodological mistakes can compromise the validity of downstream analyses (Frejno *et al.* 2025, Wen *et al.* 2025). As proteomics applications become more complex (e.g. PTM analysis, non-canonical sequences), the need for continued refinement of confidence estimation remains.

In contrast, small-molecule MS applications such as metabolomics lack comparably mature strategies for statistical validation. The high chemical diversity of metabolites, combined with a heterogeneous fragmentation behavior and limited ground-truth annotations, makes the application of target–decoy-based FDR estimation difficult. As a result, confidence measures in metabolomics often rely on heuristic thresholds or scoring metrics that lack formal statistical calibration. While there are some emerging tools toward establishing FDR strategies for metabolomics (Scheubert *et al.* 2017, Alka *et al.* 2022), these approaches have not been widely adopted yet and require further validation to reach the robustness seen in proteomics.

### 3.3 Harnessing repository-scale data for biological discovery

Public MS data repositories have become essential infrastructure for the field, supporting open data sharing, reproducibility, and secondary analyses. Initiatives aligned with the FAIR principles (Wilkinson *et al.* 2016) have led to the growth of domain-specific repositories such as PRIDE (Perez-Riverol *et al.* 2022), MassIVE, PeptideAtlas (Desiere *et al.* 2006), iProx (Ma *et al.* 2019), jPOST (Moriya *et al.* 2019), and Panorama Public (Sharma *et al.* 2018) under the ProteomeXchange umbrella (Vizcaíno *et al.* 2014) for proteomics; GNPS/MassIVE (Wang *et al.* 2016), MetaboLights (Haug *et al.* 2013), and Metabolomics WorkBench (Sud *et al.* 2016) for metabolomics, which are now beginning to coordinate under the emerging MetabolomicsHub initiative; and GlycoPOST (Watanabe *et al.* 2021) for glycomics. These resources collectively enable data reanalysis, ML development, cross-study integration, and others at scales that were previously unattainable.

One major benefit of these repositories is their role in enabling ML applications (Mann *et al.* 2021). Public datasets have become valuable resources for training models to predict molecular properties, enhancing identification and scoring in proteomics workflows. ML approaches are also increasingly applied to functional annotation tasks, such as predicting the functional potential of PTMs (Ochoa *et al.* 2020) or inferring protein characteristics from large-scale datasets (Bileschi *et al.* 2022).

Meta-analysis is another powerful use case. In proteomics, community-driven efforts, such as those coordinated by the Human Proteome Organization (HUPO), have reanalyzed thousands of datasets to characterize the human proteome, with evidence now available for approximately 93% of canonical human proteins (Omenn *et al.* 2024). These efforts span a range of objectives, from quantitative reanalysis (Choi *et al.* 2020, Dai *et al.* 2024) and PTM mapping (Ramasamy *et al.* 2020) to proteogenomics approaches for genome annotation (Levitsky *et al.* 2023), including the discovery of non-canonical proteins (Deutsch *et al.* 2024). As data volumes grow and tools mature, similar large-scale efforts are likely to expand into emerging areas such as single-cell proteomics and integrated multi-omics analyses.

In metabolomics, repository-scale reuse is also gaining momentum. ReDU provides a structured system for discovering relevant datasets across repositories based on consistent metadata, facilitating comparative analysis and integration (Jarmusch *et al.* 2020, El Abiead *et al.* 2025). At the spectrum level, tools like MASST (Wang *et al.* 2020) and Flash Entropy (Li and Fiehn 2023) allow researchers to query individual mass spectra against entire repositories to contextualize “unknowns.” In addition, the nearest neighbor suspect spectral library (Bittremieux *et al.* 2023) leverages repository-scale data to suggest candidate structures for unannotated molecules, highlighting the potential of large-scale comparisons to accelerate discovery.

Recently, standardized MS query languages have emerged to further improve accessibility and utility of public repositories. The Mass Spec Query Language (MassQL) (Damiani *et al.* 2025) offers a human-readable syntax for querying raw MS data across multiple repositories, enabling searches for spectral features such as specific precursor ions, fragment ions, and neutral losses. By abstracting over software-specific implementations, MassQL empowers researchers, including those without extensive programming skills, to mine large datasets for targeted patterns without needing to download or locally process raw files. This approach has already led to the creation of new domain-specific spectral libraries, such as a large-scale bile acid library (Mohanty *et al.* 2024), and paves the way for broad hypothesis testing and structured reanalysis across repository-scale public data.

Despite these advances, key challenges remain. Processing repository-scale MS data is computationally intensive, requiring cloud or HPC environments and well-engineered software capable of handling heterogeneous formats and metadata. Unlike smaller-scale projects, these analyses demand maintainable infrastructure and robust codebases that can scale effectively.

As discussed previously, statistical confidence is another concern. While FDR control is well-developed in individual proteomics studies, standard approaches do not readily extend to aggregated datasets, where combining datasets from diverse studies can inflate FDR and lead to an excess of false positives (Serang and Käll 2015). This issue arises because true positives tend to overlap across studies, while false positives are often unique, causing cumulative FDR inflation. Addressing this requires new statistical frameworks that account for dataset heterogeneity while maintaining confidence in discovery rates (Savitski *et al.* 2015).

Metadata quality is a further bottleneck. While experimental metadata is critical for reanalysis and interpretation, it is often inconsistently reported or embedded in unstructured formats. Efforts such as SDRF-Proteomics in proteomics (Dai *et al.* 2021), and ISA-Tab (Rocca-Serra *et al.* 2016) and ReDU (Jarmusch *et al.* 2020) in metabolomics have improved metadata standards, but adoption remains uneven. Better metadata capture at data submission, combined with automated extraction and validation tools (Claeys *et al.* 2023), could greatly improve data reuse and interoperability.

In parallel with metadata, quality control (QC) information is equally important for assessing data reliability and comparability across studies. Providing QC metrics alongside datasets supports transparent evaluation of instrument performance and experimental consistency, which are prerequisites for large-scale data integration and automated reanalysis. Recently, the HUPO Proteomics Standards Initiative (PSI) Quality Control Working Group has developed the mzQC file format, which enables standardized reporting and exchange of QC results (Bittremieux *et al.* 2017, Bielow *et al.* 2024). Incorporating both structured metadata and QC metrics at the point of data submission would substantially enhance the reproducibility, interpretability, and long-term value of public MS data resources.

Finally, the long-term sustainability of repository-scale science depends on incentivizing data sharing. Data generators often bear the burden of curation and formatting, with limited direct benefit. Yet public data are essential not only for reproducibility but also for enabling downstream reuse in various applications such as meta-analysis, benchmarking, and ML. As expectations for standardized data sharing grow, there is increasing recognition of the need to properly acknowledge contributors. Community discussions around improved citation practices, contributor recognition, and dataset-level metrics are ongoing and will be critical to sustaining high-quality data contributions.

Looking ahead, the convergence of large-scale structured data, standardized metadata, and scalable MS data processing opens the door to more autonomous modes of biological discovery using MS. Virtual laboratory concepts, where software agents reason over structured queries, metadata, literature, experimental design, and prompting from human scientists, could enable closed-loop experimentation across public and private datasets. For example, a “virtual lab” could combine spectrum-level search via tools like MASST, coupled to sample metadata via ReDU or SDRF, and AI-assisted summarization to propose new experimental hypotheses or suggest orthogonal validation experiments. These ideas are increasingly plausible as workflows become more digitized and automated; indeed, efforts toward “lights-out” sample preparation robotics (Schär *et al.* 2025) provide the physical infrastructure for seamless, automated experimental execution, while computational infrastructure for repository-scale access and metadata standardization enable contextual reasoning.

### 3.4 Multi-omics integration for deeper biological insights

Integrating data across multiple omics layers offers the potential for a more holistic understanding of biological systems by linking genomic, transcriptomic, proteomic, metabolomic, and other molecular profiles (Hasin *et al.* 2017, Karczewski and Snyder 2018). While single-omics studies provide valuable insights within their respective domains, multi-omics approaches aim to uncover relationships across molecular layers, such as how genetic variation influences protein expression or how metabolic states reflect transcriptional regulation (Wörheide *et al.* 2021). However, many current applications of multi-omics remain limited in scope, often focusing on simple overlap analyses of top features rather than truly integrative modeling.

Effective multi-omics integration requires methods that can capture dependencies and interactions between omics layers in a statistically sound and biologically meaningful way. Such approaches have the potential to improve biomarker discovery, refine mechanistic hypotheses, and provide context-specific insights into disease or treatment responses. However, realizing these benefits requires moving beyond concatenated datasets and toward analytical frameworks that explicitly model relationships across layers.

**Figure 2.**
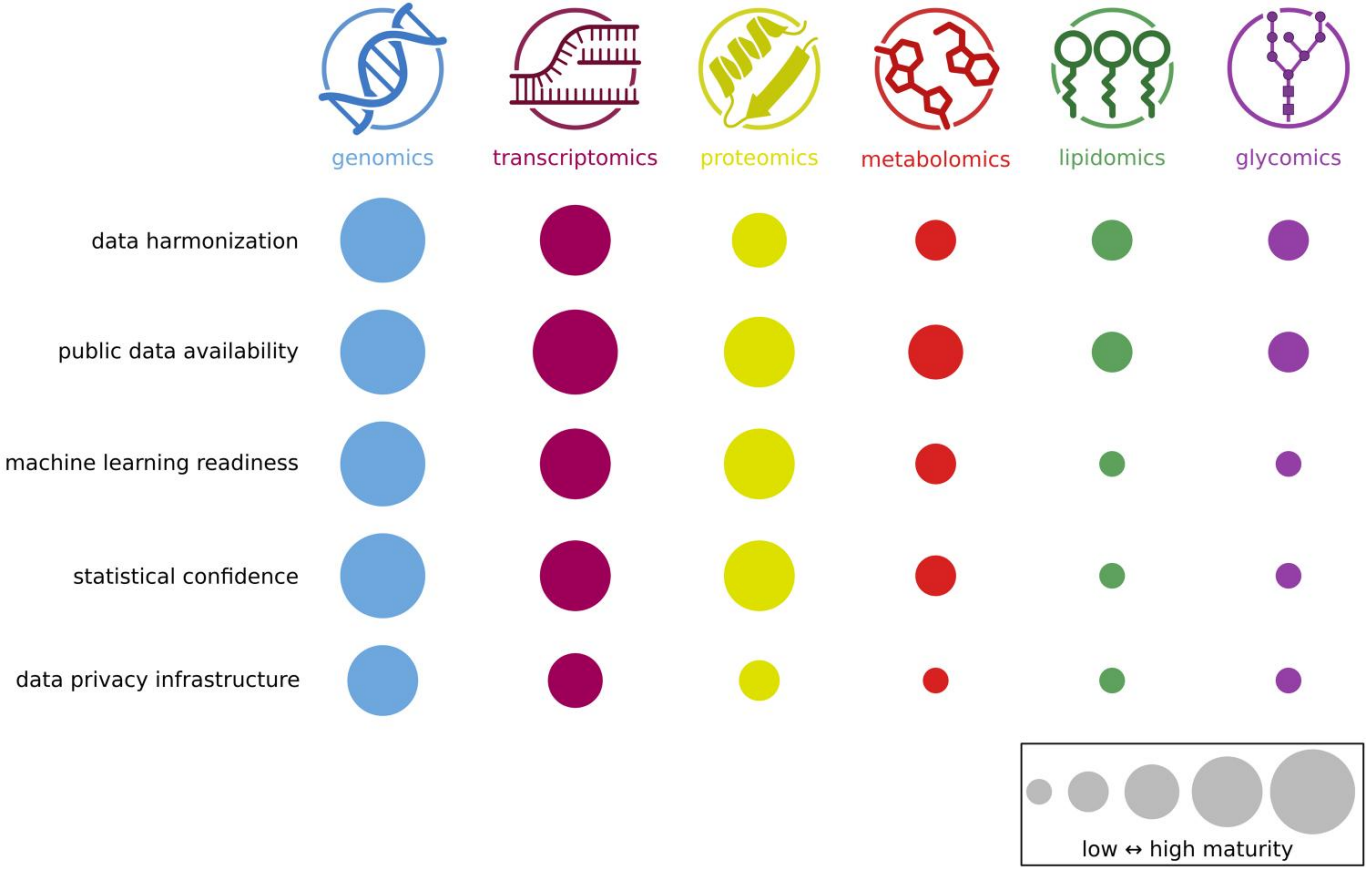
Relative computational maturity of different omics domains. Genomics and transcriptomics lead in maturity, reflecting well-established standards and infrastructure. Proteomics occupies an intermediate position, while metabolomics, lipidomics, and glycomics still face substantial challenges across several dimensions. Uneven maturity across omics fields contributes to the difficulties of multi-omics integration. Circle size represents relative maturity, from low (small) to high (large).

Several challenges impede the development of robust multi-omics integration methods (Fig. 2). Omics data types differ in their dynamic ranges, measurement noise, missingness, and resolution, complicating direct alignment and joint modeling. A further practical obstacle is the lack of consistent identifier mapping across omics layers—for example, between genes, proteins, metabolites, and pathways—which hampers accurate data linkage and biological interpretation. Standard pipelines for normalization and integration across omics types remain underdeveloped, and most existing tools are designed for single-modality workflows. Moreover, many statistical models lack mechanisms to account for variation in data quality between layers, leading to biased or overconfident conclusions when integrating heterogeneous datasets.

Beyond traditional omics layers, structural proteomics approaches such as cross-linking mass spectrometry (XL-MS) (O’Reilly and Rappsilber 2018) and hydrogen–deuterium exchange (HDX) (Konermann and Scrosati 2024) offer complementary insights by probing the spatial organization and dynamics of proteins and protein complexes. These techniques are increasingly integrated into multi-omics workflows to add a structural dimension to systems-level analyses. The introduction of high-accuracy structure prediction tools such as AlphaFold (Jumper *et al.* 2021) has dramatically expanded our ability to model protein structures *in silico*. These predicted structures now serve as valuable priors for interpreting XL-MS and HDX data, facilitating more accurate cross-link mapping and conformational analysis (Drake *et al.* 2022). Conversely, experimental data from XL-MS can validate or constrain *in silico* predictions, and even guide refinement of structural models in regions of low confidence (Stahl *et al.* 2023). Integrating such structural information into multi-omics analyses may reveal mechanistic insights that are inaccessible through sequence-based data alone, such as conformational regulation, allostery, or the physical basis of protein–protein interactions.

Broader visions are also emerging in systems biology, such as the ambition to construct an AI-powered “virtual cell”: a multi-scale, multi-modal neural architecture capable of simulating molecular, cellular, and tissue-level behavior across diverse biological states (Bunne *et al.* 2024). MS data, especially from public repositories enriched with high-quality annotations and cohort-level metadata, provide a crucial training set for such efforts (Sun *et al.* 2025). Unlike sequencing-based technologies, MS uniquely captures diverse molecular species, including proteins, lipids, metabolites, and glycans, that are essential for accurately modeling biological complexity at multiple levels.

Ultimately, the integration of multi-omics data represents an essential goal in computational biology, promising to unlock new layers of understanding in complex biological systems. Achieving this goal will require overcoming significant challenges in data compatibility, statistical modeling, and computational resource allocation.

### 3.5 Building machine learning literacy for mass spectrometry

ML and AI have become increasingly central to computational MS, enabling advances in spectrum interpretation, molecular property prediction, quality control, and data integration, among other topics. However, effectively applying ML to MS data remains challenging. The complexity of MS datasets requires careful methodological choices and a strong understanding of both domain-specific and computational principles.

While ML tools are becoming more accessible—for example, through software libraries like Scikit-Learn (Pedregosa *et al.* 2011) and various deep learning frameworks—this accessibility can mask underlying complexities. Without sufficient training in ML fundamentals, users may select inappropriate models, overlook preprocessing needs, or apply flawed evaluation strategies (Adams and Bittremieux 2025). In MS contexts, where the structure of the data often reflects subtle biological phenomena, such missteps can lead to incorrect conclusions or irreproducible findings. Model selection, hyperparameter tuning, and evaluation metrics must be tailored to the specific characteristics of MS data to ensure meaningful results.

A particularly critical issue in omics-based ML is the risk of data leakage, where information from outside the training set inadvertently informs model training, resulting in overestimated performance. This can occur through improper data splitting (Desaire 2022), reusing test data during model optimization, or embedding confounding factors in input features. Data leakage often goes unnoticed, even in published studies (Quinn 2021), and undermines trust in reported outcomes. Overfitting is another common challenge, particularly given the large number of features relative to sample size in many omics datasets. Regularization, robust cross-validation, and careful feature selection are essential but must be adapted to the nuances of MS data, including frequent missing values and noise (Adams and Bittremieux 2025).

Transparent reporting is key to enabling reproducibility and scientific rigor. ML-based MS studies should clearly describe preprocessing steps, model architecture and parameters, validation strategies, and performance metrics, and where possible, register models in dedicated resources such as the DOME (Data, Optimization, Model, and Evaluation) (Walsh *et al.* 2021) registry to ensure standardized documentation and accessibility (Palmblad *et al.* 2022). As models grow more complex, particularly with the adoption of deep learning, such documentation becomes even more important for peer evaluation and reuse.

Addressing educational gaps is also essential. Many researchers applying ML to MS come from biology or analytical chemistry backgrounds and may not have formal training in statistical learning. Core concepts such as overfitting, cross-validation, and proper data partitioning are frequently underappreciated. To address this, hands-on training opportunities, such as workshops, tutorials, and summer schools, are increasingly being offered within the MS community. These initiatives aim to equip researchers with practical skills and promote best practices in ML application (Rehfeldt *et al.* 2023).

Finally, interdisciplinary collaboration remains one of the most effective ways to ensure methodological rigor. Involving data scientists and statisticians early in project planning helps align study design with appropriate computational analysis, prevents common pitfalls, and fosters development of more robust and interpretable models. Likewise, involving biologists and chemists throughout an analysis helps ensure that computational frameworks and analyses are grounded and interpretable. As MS increasingly intersects with advanced computational methods, fostering this cross-domain dialogue will be essential for responsible and impactful research.

### 3.6 Privacy challenges in clinical omics

As MS becomes increasingly integrated into clinical research, the handling of sensitive human data raises important ethical and privacy considerations (Mann *et al.* 2021). While public data sharing is vital for reproducibility and scientific progress, clinical datasets, particularly those containing individual-level health or molecular information, require careful management to ensure data privacy is not compromised.

Privacy risks in proteomics share parallels with those in genomics (Lin *et al.* 2004). Protein sequences, derived from expressed genes, can carry identifying features, particularly when combined with phenotypic or clinical metadata. Although proteomic data lacks certain elements found in genomic sequences, such as non-coding regions, variations in protein-coding regions or characteristic expression patterns may still allow for re-identification under certain conditions (Geyer *et al.* 2021). Metabolite, lipid, and glycan expression patterns potentially carry identifiable information as well (Keane *et al.* 2021). These risks are amplified in the context of multi-omics datasets, where linking across molecular layers can increase the resolution of individual profiles.

Regulatory frameworks such as the General Data Protection Regulation in the European Union and the Health Insurance Portability and Accountability Act in the United States impose strict requirements for managing sensitive personal data. These regulations mandate stringent technical and organizational measures to protect the privacy of the affected persons. However, public MS repositories, originally designed to promote open access and broad reuse, currently lack the infrastructure for secure authentication, access control, or compliance tracking (Bandeira *et al.* 2021). As a result, it becomes difficult to share clinically sensitive data in a privacy-compliant manner, creating obstacles for researchers who wish to adhere to the FAIR data principles while protecting individual privacy. Without privacy-aware repositories or controlled-access solutions, clinical proteomics—and increasingly, clinical metabolomics and lipidomics—risks falling behind in data sharing. This challenge needs to be addressed urgently in order to ensure that the positive role data sharing has been playing in MS-based omics can continue in clinical settings as well. Ideally, the MS community can learn from—and align itself with—the efforts in clinical genomics, where suitable repositories have been developed (D’Altri *et al.* 2025).

In parallel to repository infrastructure development, privacy-preserving computational techniques offer complementary solutions. Federated learning, where ML models are trained across decentralized datasets without transferring the raw data, or approaches based on homomorphic encryption are particularly well suited for clinical MS applications (Burankova *et al.* 2025, Cai *et al.* 2025). These approaches require sophisticated infrastructure, but they allow multi-institutional analyses while maintaining local control over sensitive data.

### 3.7 Role of the CompMS community of special interest

ISCB’s CompMS Community of Special Interest brings together researchers from computer science, bioinformatics, statistics, and molecular biology to advance the analysis and interpretation of MS data. Uniting disciplines under a shared interest in MS-based omics, the CompMS community supports proteomics, metabolomics, lipidomics, glycomics, and related fields where MS serves as a core analytical platform. Through its activities, the community fosters collaboration and knowledge exchange aimed at transforming complex datasets into meaningful biological insights.

**Figure 3.**
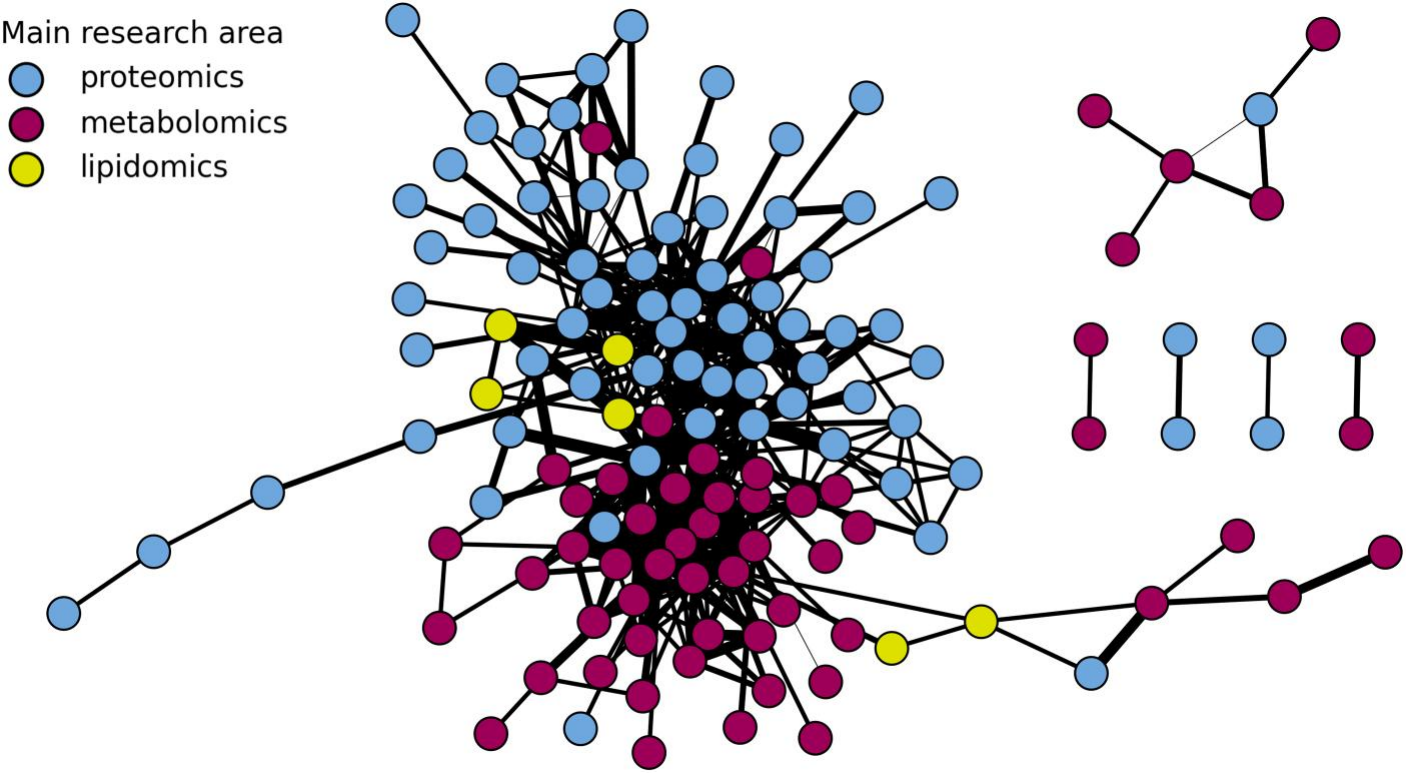
Co-publication network of users from the CompMS Slack workspace. Nodes represent individual users (based on best-effort name matching to PubMed), colored by their primary research area as inferred by PubMed keyword matching. Edges indicate co-authorship of at least one scientific publication, with edge width reflecting the number of shared publications. The network highlights the collaborative and interdisciplinary nature of the CompMS community, bridging proteomics, metabolomics, lipidomics, and related fields.

CompMS emphasizes community building and open exchange through accessible communication channels. A central hub for this interaction is the CompMS Slack workspace (https://compms.slack.com), where researchers at all career stages seek advice, share software updates, and discuss emerging challenges and opportunities in computational MS (Fig. 3). This environment promotes open dialogue and collaborative problem-solving, encouraging both technical innovation and mentorship.

The community’s primary annual event is the CompMS track at the Intelligent Systems for Molecular Biology (ISMB) conference, which showcases the latest advances in computational MS, including method development, bioinformatics, and ML applications. These sessions draw a multidisciplinary audience and provide a venue for presenting novel tools and addressing key challenges across MS-based domains. By facilitating cross-disciplinary interaction, the CompMS track serves as a vital forum for the exchange of ideas and development of shared standards. Beyond ISMB, CompMS actively engages with the broader MS community by contributing to Bioinformatics Hub sessions at the annual conferences of the American Society for Mass Spectrometry (ASMS) and HUPO, and by seeking synergies with initiatives led by organizations such as HUPO, the HUPO-PSI, the Metabolomics Society, the European Bioinformatics Community for Mass Spectrometry (EuBIC-MS), and ELIXIR. Such cross-community involvement helps to bridge the gap between computational and experimental MS communities, connecting developers of algorithms and data standards with researchers driving new MS technologies and applications. By facilitating collaboration across these diverse forums, CompMS provides a platform for advancing computational MS globally.

A distinctive feature of the CompMS community is its strong support for early-career researchers. The ISMB track highlights junior contributions through talks, posters, and awards, while dedicated travel fellowships broaden participation by enabling students and postdocs to attend. These opportunities help integrate emerging researchers into the field and support the development of a vibrant, inclusive community committed to advancing computational MS.

## 4 Conclusions

MS has become a foundational technology in the life sciences, supporting deep molecular analyses across proteomics, metabolomics, lipidomics, glycomics, and other omics disciplines. Its ability to detect and characterize a broad range of molecules makes it indispensable for understanding biological systems in both health and disease. As research increasingly relies on high-throughput, high-resolution, and multi-dimensional data, the role of MS in capturing phenotypic complexity continues to grow.

This perspective outlined recent advances in computational MS, including developments in acquisition strategies, scalable data processing, and ML/AI. We also highlighted ongoing challenges, such as batch effects and data harmonization, the need for robust statistical confidence across domains, limitations in multi-omics integration, and emerging concerns around privacy in clinical applications. Addressing these issues is critical to ensure that MS data remains reliable, interpretable, and broadly reusable.

Looking ahead, realizing the full potential of computational MS will depend on developing systems that are not only scalable and interoperable but also “AI-ready.” In this context, AI-readiness refers to the extent to which MS (and derived) data, metadata, and analytical tools are structured and harmonized to enable ML and automated reasoning. Concretely, this includes the use of FAIR principles, standardized metadata schemas, transparent and reproducible preprocessing pipelines, and programmatic data access through machine-actionable interfaces. Establishing such foundations will be essential for enabling autonomous data analysis, knowledge discovery, and virtual laboratory environments in the future.

To further advance MS data toward AI-readiness, a new AI Working Group has recently been established under the HUPO-PSI (https://www.psidev.info/ai-readiness) (Deutsch *et al.* 2023). The group’s goal is to make public proteomics data suitable for AI applications and to support and standardize the incorporation of AI within workflows that generate new data. This is a timely, community-driven effort, open to all interested scientists, and represents an important step toward enabling reproducible and AI-augmented discovery in MS-based related omics disciplines.

These emerging standardization and interoperability efforts complement the broader activities of the CompMS Community of Special Interest. By fostering collaboration across disciplines, advancing methodological development, and supporting early-career researchers, the CompMS community is helping to shape a robust and forward-looking computational MS ecosystem that will continue to empower discovery across diverse biological domains.
